# The Research on Organizational Justice in Scopus Indexed Journals: A Bibliometric Analysis of Seven Decades

**DOI:** 10.3389/fpsyg.2021.647845

**Published:** 2021-06-10

**Authors:** Muhammad Irfan Sheeraz, Ungku Norulkamar Ungku Ahmad, Muhammad Ishtiaq Ishaq, Muhammad Sarfraz, Khalil Md. Nor

**Affiliations:** ^1^Azman Hashim International Business School, Universiti Teknologi Malaysia, Johor Bahru, Malaysia; ^2^School of Management Sciences, Quaid-i-Azam University, Islamabad, Pakistan

**Keywords:** organizational justice, bibliometric analyses, Scopus database, VOSviewer, distributive justice

## Abstract

The organizational justice terminology has had a long journey to become one of the significant contributors to organizational success. Recently, an intense global upsurge in the use of organizational justice terms in publications has forced us for this bibliometric analysis in order to look at the overall publications on organizational justice. The objective of the current research is to advance knowledge about organizational justice research trends using Scopus database and bibliometric analysis research. The analysis was performed to see the publication trends between the years 1941 and 2018; it used authors, journals, countries, academic discipline, research institutes/universities, and various keywords related to organizational justice as search words. After careful consideration and using multiple checkpoints for eliminating irrelevant studies, 5,650 research articles were analyzed. In the realm of organizational justice, procedural justice was the most frequently occurred among other dimensions. Moreover, variables such as organizational trust, job satisfaction, organizational commitment, citizenship behavior, ethics, and turnover are major concepts that occurred within organizational justice research. Some variables with infrequent occurrences, along with future recommendations and study limitations, are also discussed.

## Introduction

Justice is an important matter in organizational life and is a fundamental feature in human behavior (Adams, 1965). A wide range of scholars, including psychologists, political scientists and managers, have paid significant attention to the topic (Melkonian et al., 2011). Many religious books like the *Holy Quran*, the *Bible*, the *Bhagavad Gita*, and the *Granth* also stress the importance of justice in every matter of social life as well as official life (see The Quran, 16:91; The Bible Hosea 12:6; The Bhagavad Gita, 4:7–8; The Granth, p. 308). Organizational justice has been identified as one of the most frequently studied topics in various disciplines, such as organizational behavior, organizational psychology and human resource management (Colquitt et al., 2001; Lu and Guy, 2018). Organizational justice is a significant research area in the realm of organizational behavior, as highlighted in an important bibliometric analysis by Piotrowski (2014, 2016).

The term “organizational justice” was first coined by French (1964) to describe the employee's perception of fairness in an organization. Organizational justice refers to the fairness of a decision an organization makes, the procedure they use in making decisions and the interpersonal treatment employees receive (Wan, 2016). It is a vital element in shaping employees' behavior and attitude, and it is the intangible glue that allows employees to work together effectively and efficiently (Colquitt and Rodell, 2011; Rupp et al., 2017). The past 30 years have seen increasingly rapid advances in the field of organizational justice and a considerable amount of literature has discussed the importance of organizational justice (Rupp et al., 2017; Krishnan et al., 2018). This massive increase in the literature of organizational justice has forced us to investigate the present trend of research on the topic. Although extensive research has been carried out on organizational justice, these systematic literature reviews (Rovenská, 2017; Virtanen and Elovainio, 2018; Wright and Nyberg, 2018), meta-analyses (Colquitt et al., 2013; Vaerenbergh et al., 2018), and bibliometric analyses (Johnson, 2009) are restricted to limited areas. These reviews and meta-analysis studies focus on certain concepts and revolve around certain predefined criteria, whereas Johnson's (2009) study was limited to one subject area.

## Methodology

**Figure 1:**
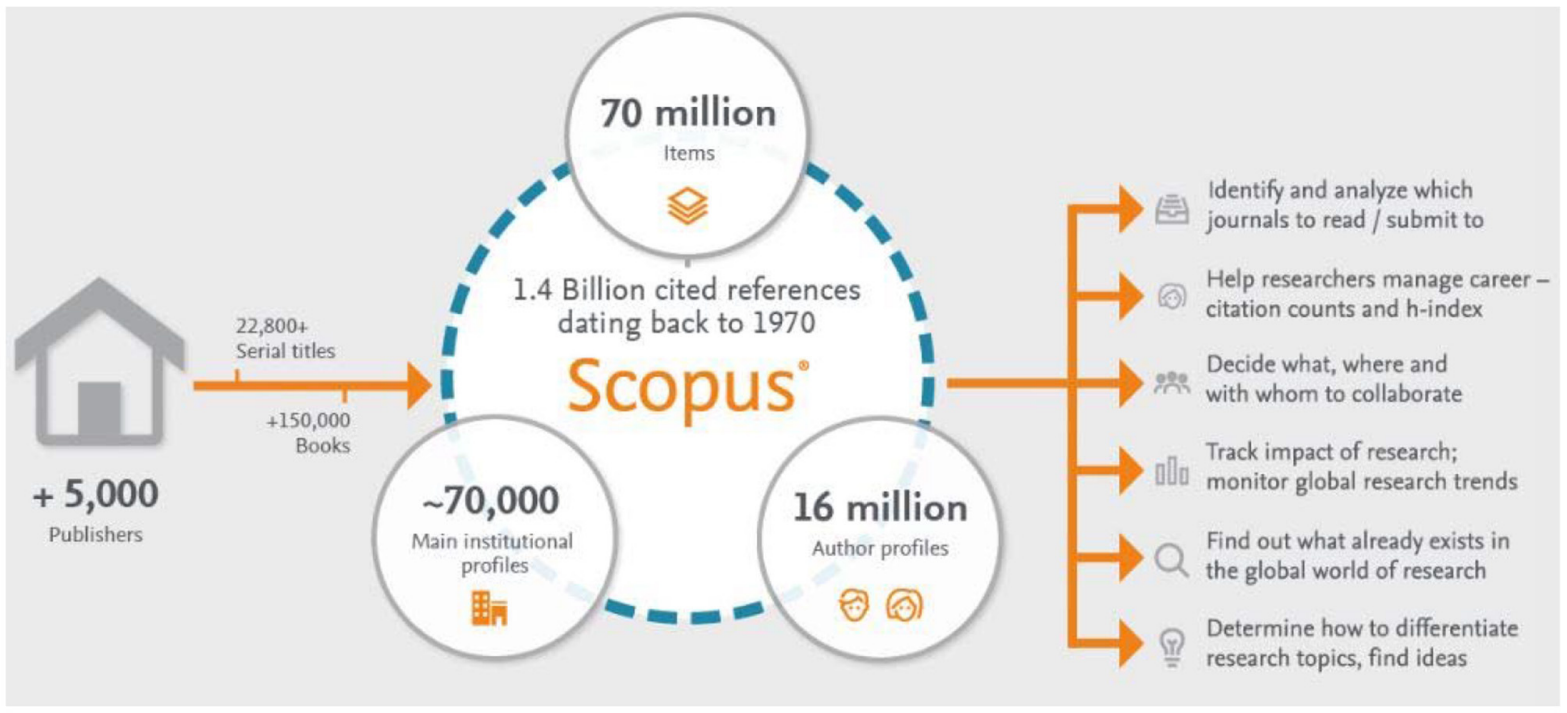
Scopus at a Glance (Source: Scopus, 2019).

The academic literature has proposed various approaches to examining the influence of specific variables, such as scientometrics, bibliometrics, informetrics, webometrics, librametrics, patentometrics, altmetrics, and article-level metrics (Das, 2015). Bibliometric data analysis helps researchers to do a comprehensive investigation of a variable from various angles and highlights its development path (Fellnhofer, 2019). Therefore, this study used bibliometric analysis to investigate the significance of organizational justice in academic research. This analysis technique offers multiple ways to understand the variable under investigation: (1) it develops our understanding on a particular research area by giving insights about the field of research, variable behaviors and its regularities; (2) it reveals recent trends about the variable; and (3) it provides the relationships and networks of the variable. We used the Scopus Database for this research, since it indexes the best journals with the most recent articles (Aghaei Chadegani et al., 2013). In addition, Scopus is the largest abstract and citation database; it provides more accurate data (Franceschini et al., 2016) and deals with 1.4 billion citations and 16 million authors' profile, as shown in Figure 1. These features make Scopus the right choice for the bibliometric analysis of such an extensive research variable as organizational justice.

### Data Source and Data Mining

**Figure 2:**
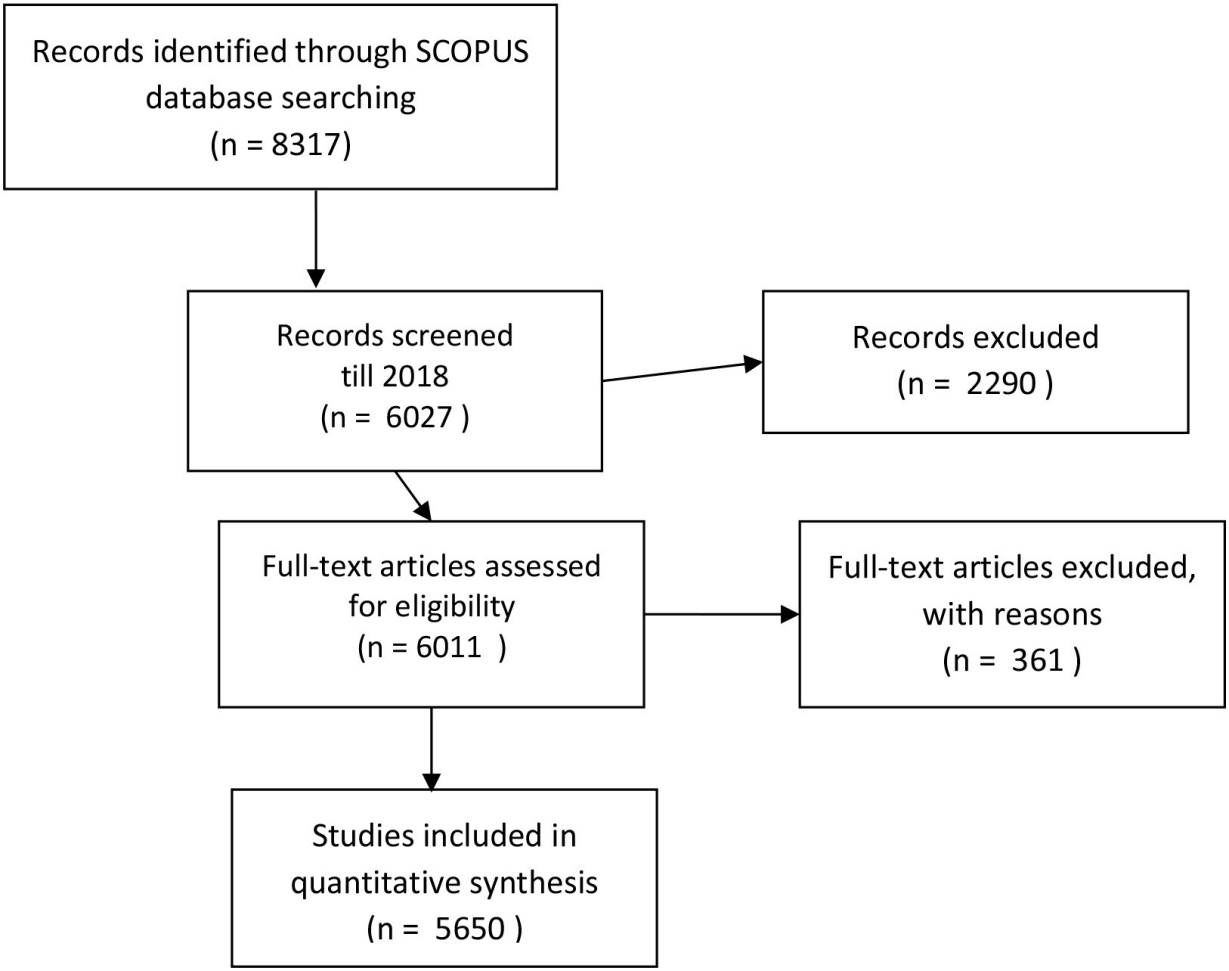
Process of article search and number of articles.

The present study was designed to determine the research trends concerning organizational justice. The search used the keyword of “organizational justice,” along with its dimensions, and was performed for articles, abstracts, and keywords. For this purpose, the data were gathered during the 1st week of September 2019 using the Elsevier Scopus database at various time points. The initial results in the first step revealed 8,317 articles; the oldest article on organizational justice was published in 1941, whereas 463 articles were published in 2018. We limited our research until 2018, therefore, in the second step we found 6,027 articles. In step three, 361 articles were excluded from the list, as they were either review/conceptual or meta-analytical articles. Lastly, Valderrama-Zurián et al. (2015) has argued that papers are duplicated on the Scopus database. We checked the electronic identifier (EID) of the articles systematically and could not find any duplication of the articles. However, manual check-up identified 16 duplicate articles. These articles were finally removed at step four. Hence, a total of 5,650 articles were used in the final data analysis, which is step five. The process of data collection is provided in Figure 2, whereas the search strings are available in Appendix A in Supplementary Material.

### Bibliometric Maps

The author details, affiliated country, and keywords of 5,650 articles were exported to VOSviewer, a software tool for constructing and visualizing bibliometric webs. The VOSviewer is especially used when working with small and large datasets; it displays data maps and various analytical analyses (Kokol et al., 2018; Md Khudzari et al., 2018; Llanos-Herrera and Merigo, 2019; Shah et al., 2019). Similarly, Van Eck and Waltman (2019) stated VOSviewer provides additional mapping methods based on scientific principles for creating useful maps, networks and data. Thus, all of the maps combining the respective linkage groups that were created using the VOSviewer include items. In this research, the items are a combination of entities of interest, namely authors, keywords, articles or the author's affiliated country, as defined by the VOSviewer. The author keywords provide information about research trends from the point of view of the researchers and have proved to be important in monitoring the development of the field. Between a pair of items, there can be a link or connection. Each link has a strength, represented by a positive numerical value; the highest numerical value indicates the link strength and vice versa. Moreover, VOSviewer map does not display two items at a time for example, both country and keywords.

Also, in the case of co-authorship analysis, the link strength between countries indicates the number of co-authored articles publications with the affiliation of more than one country. In the analysis of co-authorship network, we included all 99 countries affiliated with the 5,650 publications. The affiliated countries were clustered into four continents and one region—Asia, America, Africa, Europe, and Oceania region—for further analysis of this study. The total link strength indicates the total strength of the co-authorship links of a given country with other countries. We also presented the countries' network of published documents graphically with the help of the VOSviewer software.

In addition, in the case of co-occurrence analysis, the link strength between author keywords indicates the number of publications in which two keywords occur together. The analysis of co-occurrence of author keywords involved 390 keywords from 5,650 articles. Before importing the list of author keywords to the VOSviewer, synonymic single words and analogous phrases were analyzed manually. For example, adolescents and young adults all counted as adults and were re-labeled as such. Similarly, OCB, citizenship behavior, and altruism or organizational citizenship behavior were all re-labeled as organizational citizenship behavior. The same method was used with all the terms which were used interchangeably in the publications. The trend of research outputs between the central theme (keyword co-occurrences) and its dimensions (in total publications) was compared too. For example, fairness, justice, and social justice were all included. It may also to note that we have used English small alphabet “n” to refer to “number of occurrences.”

## Results and Discussions

### Publication Output and Growth of Research Interest

**Figure 3:**
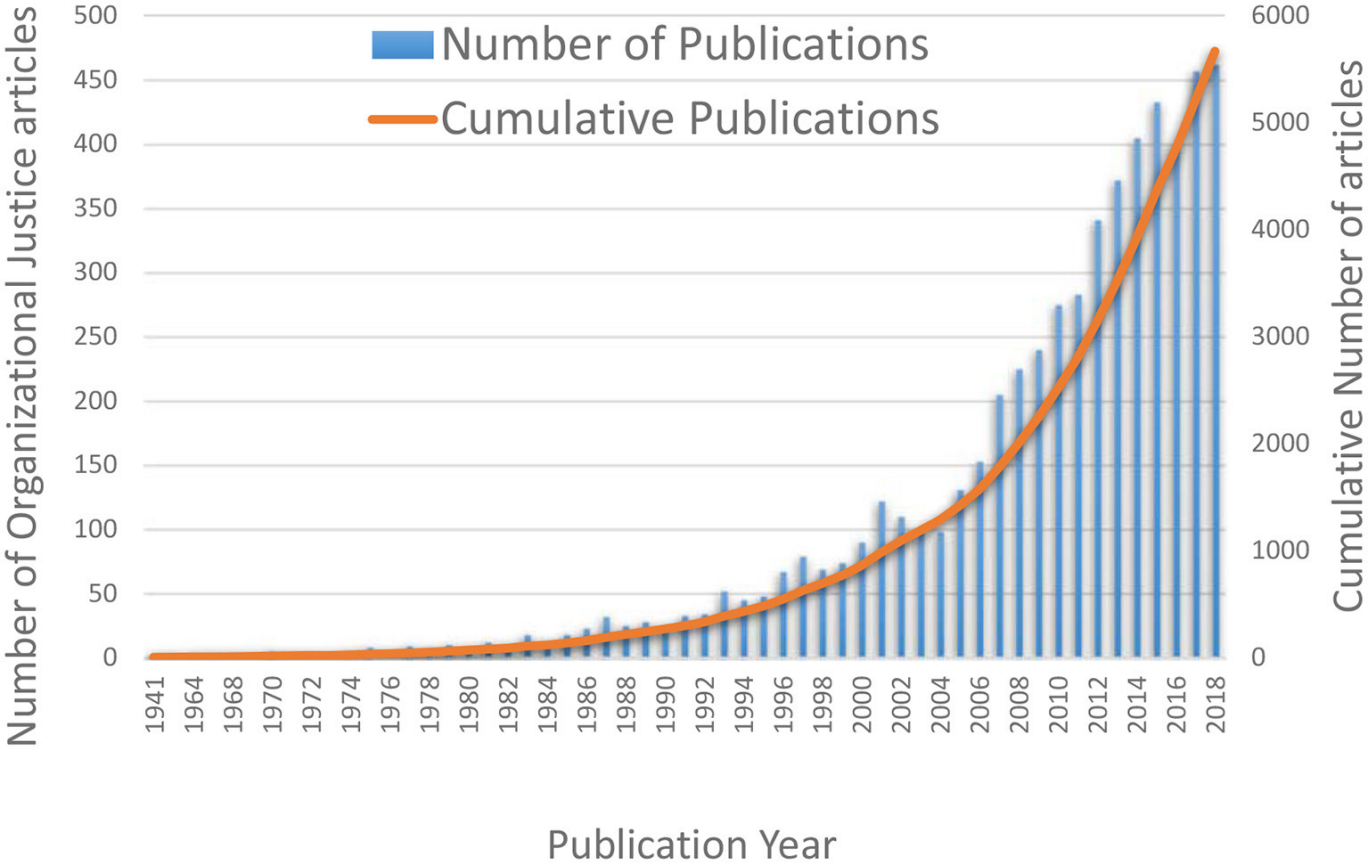
The year-wise and cumulative number of articles on organizational justice research.

Between 1941 and 2018, 5,650 research articles related to organizational justice were published (Figure 3). Among these 5,650 publications, only 432 are available with open access. The oldest publication dates back to 1941, and there was no other publication until 1953. The articles crossed the 100 publications barrier in 1983. One major reason for the gap in early periods may be due to Second World War which affected almost every field of the world including research. By the end of the twentieth century, a steady increase in publications had emerged in the academic literature. Also, 60% of the articles were published after 2009. We believe that the massive increase in publications is due to the increase in higher education institutions (Chen et al., 2009), and collaborations, the rise of the research culture in some countries (such as Pakistan) as well as an overall increase of publications across the globe (Nature, 2018; Researchtrends, 2019) From these statistics, one can assume that the number of publications will continue to rise significantly in the coming years.

#### Subject Areas in Organizational Justice Research

Organizational justice has an equal concern for employers and employees and is not limited to any particular field, as mentioned in Table 1. Almost all subject areas, such as social sciences, business, engineering, environment, and medicine, have investigated organizational justice. The Scopus database has categorized these 5,650 articles into 27 subject areas. Table 1 presents the top 10 subject areas on organizational justice research. In organizational justice research, the social sciences have the highest number of publications, whereas business, management, and finance have the second highest number. It is often assumed that organizational justice relates most to psychology, but our analysis shows that psychology falls to third place. We noticed that the number of articles categorized in these 27 subject areas is far more than our search results. Although there are substantial differences among the fields of study (Franceschini and Maisano, 2014), we realized that journals are sometimes categorized in more than one field. For example, the *Journal of Managerial Psychology* is categorized into four fields: business, management, and accounting; psychology (social); decision sciences; and psychology (applied). Thus, we found that an article published in interdisciplinary journals, which are categorized in more than one field, has more scope and citations than the articles published in a journal categorized in only one field, e.g., *Marketing Letters*, the *European Journal of Marketing*, the *Journal of Organizational Behavior*, and the *Annals of Finance* (Smolinsky, 2016).

**Table 1:** Number of publications subject-wise.

**Rank**	**Subject area**	**TP^a^**	**JHP^**b**^**	**TP**
1	Social Sciences	2,752	Social Justice Research	155
2	Business, Management, and Accounting	1,881	Journal of Business Ethics	91
3	Psychology	1,525	Journal of Applied Psychology	106
4	Arts and Humanities	895	Journal of Business Ethics	87
5	Medicine	789	Criminal Justice and Behavior; Policing	31 each
6	Economics, Econometrics, and Finance	560	Journal of Business Ethics	91
7	Environmental Science	280	Energy Policy	17
7	Decision Sciences	212	Journal of Managerial Psychology	43
9	Nursing	193	Journal of Medical Ethics	24
10	Computer Science	137	Computer and Human Behavior; Theory and Decision	9 each

a *TP, Total publications*.

b *JHP, Journal with highest publications*.

#### Languages of the Articles

Organizational justice research is multilingual as we found articles in various languages. Our results show that the articles used in this study were published in 24 languages. About 94.35% of articles were published in English followed by 0.79% in Spanish and 0.77% in German, whereas 11 languages, including Dutch, Korean and Arabic, had fewer than five articles published. According to a condition imposed by most of the publishers, the author must have an abstract in English even if it is written in another language, which also enhanced the range of the Scopus database. We also noticed that top 10 journals listed in Table 2 were published in English, so their coverage is much broader than journals published in other languages, such as *Notizie di Politeia*, which is published in Italian. However, we included all articles published in any language as the data gathered are based on title, abstract, and keywords.

**Table 2:** Top 10 most productive journals.

**Rank^a^**	**Journal**	**TP^**b**^**	**TGC^**c**^**	**CiteScore**	**MCA^**d**^**	**TGC**	**Publisher**
1	Social Justice Research	155	3,643	1.57	The role of procedural and distributive justice in organizational behavior	409	Springer Nature
2	Journal of Applied Psychology	105	16,115	6.86	On the dimensionality of organizational justice: A construct validation of a measure	1,945	APA
3	Journal of Business Ethics	91	2,063	4.46	To share or not to share: Modeling tacit knowledge sharing, its mediators and antecedents	196	Springer Nature
4	Journal of Organizational Behavior	62	5,604	6.59	Trust as a mediator of the relationship between organizational justice and work outcomes: Test of a social exchange model	695	Wiley-Blackwell
5	Journal of Applied Social Psychology	55	1,016	1.99	The Effects of Procedures, Social Accounts, and Benefits level on Victims' Layoff Reactions	101	Wiley-Blackwell
6	Intercountryal Journal of Human Resource Management	54	1,560	2.71	The contribution of corporate social responsibility to organizational commitment	358	Taylor & Francis
7	Organizational Behavior and Human Decision Processes	51	4,312	3.82	The mediating effects of social exchange relationships in predicting workplace outcomes from multifoci organizational justice	357	Elsevier
8	Journal of Managerial Psychology	43	1,854	2.05	Antecedents and consequences of employee engagement	1,086	Emerald
9	Journal of Business and Psychology	37	861	3.17	Fairness reduces the negative effects of organizational politics on turnover intentions, citizenship behavior and job performance	83	Springer Nature
10	Journal of Management	36	2,575	10.96	Fairness perceptions and trust as mediators for transformational and transactional leadership: A two-sample study	484	SAGE

a *Rank, By total number of publications*.

b *TP, Total publications*.

c *TGC, Total global citations till 2018*.

d *Most cited article*.

#### Funding of Research Publications

The data reveal that about 13% of the research projects are sponsored by various universities, ministries, research institutions and organizations. Among these funded agencies, National Natural Science Foundation of China and the National Science Foundation in the USA finance 43 publications each. The Australian Research Council subsidizes 42 publications. However, China is the leader in financing organizational justice research; it funds about 20% of all publications, whereas the USA funds <2%. Future researchers should keep in mind that although there is funding, but it is limited for the field of organizational justice research.

### Top Journals

Our analysis shows that the top 10 most productive journals are maintained by seven different well-known publishers (Table 2). Among these top 10 journals, only one is published by a psychological association—the American Psychological Association—whereas the rest of the journals are being produced by professional publishers. Among these journals, three are being published by Springer Nature and two by Wiley-Blackwell. We noted that the coverage of the top journals is low, as the total number of journals is high, i.e., 5,650 articles published in 1,914 journals indexed by the Scopus database.

Article recognition is determined by the number of post-publication citations by other researchers (Baltussen and Kindler, 2004). The *Journal of Applied Psychology* is a highly cited journal. Moreover, one article from this journal also has the highest number of citations amongst all 5,650 articles. The second highest most productive is the *Journal of Organizational Behavior*, but one of its articles is the third highest cited article, with a total number of 695 citations. The *Journal of Managerial Psychology* is the eighth most cited journal, but one of its articles has the second highest number of citations. The range of total citations by any journal is from 1,086 to 16,198 whereas the range of the individual highly cited articles was from 83 to 1,946 of these journals.

Moreover, the Scopus database measures the average number of citations for the articles through CiteScore, which is released once a year. CiteScore can influence the decision of authors to select the journal that most fit their research work for publication. According to the 2018 CiteScore, only three journals have a CiteScore above five. The highest CiteScore was received by the *Journal of Management*, with a score of 10.96, whereas the *Journal of Applied Psychology* and the *Journal of Organizational Behavior* are second and third, respectively. This CiteScore is not limited to only the field of organizational justice research. We also noted that *Social Justice Research journal* was ranked first due to its high number of publications, but it has a CiteScore below two, which shows the journal is not frequently cited. We have gathered the list of the top 31 CiteScore journals having over 20 articles on organizational justice in Appendix B in Supplementary Material. We hope that it will be helpful for researchers to find possible journals to submit their organizational justice research work.

**Figure 4:**
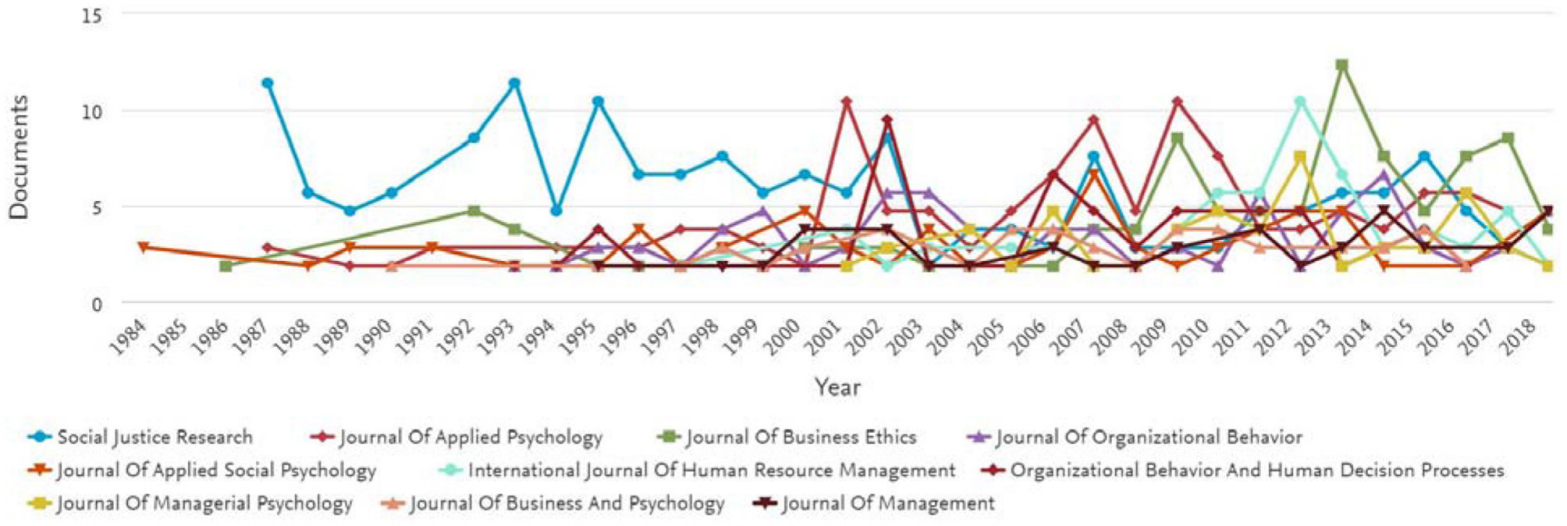
Source journals with publication trends from 1984 to 2018.

If we look closely into the publication trend for these top 10 journals, we find that the Scopus database has provided analysis from 1984 to 2018. In the beginning, the *Social Justice Research Journal* was publishing more articles followed by the *Journal of Business Ethics*. After the turn of the twenty-first century, the *Journal of Applied Psychology*, the *Journal of Organizational Behavior*, and *Human Decision Processes* took the lead. Recently, the *International Journal of Human Resource Management* and the *Journal of Business Ethics* have published more articles. However, most recently, the *Journal of Business Ethics* has become the first choice for researchers publishing on organizational justice research, whereas the rest of the journals have maintained the same publishing ace, i.e., an average five articles per annum (Figure 4). Moreover, the *Journal of Business Ethics* has published the most articles for 1 calendar year, which was 12 in 2013.

### Top 10 Highly Cited Articles

Table 3 presents the data for the top 10 most cited articles on the Scopus database. Furthermore, among the top 10 most cited articles, the *Journal of Applied Psychology* is leading with four articles. However, no other journal has more than one article. It is also pertinent to note that the majority of these highly cited articles have been published in journals related to psychology. All these articles have been published by the authors of western countries. Among these 10 articles, not surprisingly, 13 of these authors are affiliated with USA, whereas one is from Sweden and two are from Canada.

**Table 3:** Top 10 highly cited research articles.

**Rank^**a**^**	**Title**	**Authors**	**Citations^**b**^**	**Source and year**
1	On the dimensionality of organizational justice: A construct validation of a measure	Colquitt, J.A	1,945	Journal of Applied Psychology, 86(3), pp. 386-400, 2001
2	What firms do? Coodination, identity, and learning	Kogut, B., Zander, U.	1,666	Organization Science, 7(5), pp. 502-518, 1996
3	Relationship between organizational justice and organizational citizenship behavior: Do fairness perceptions influence employee citizenship?	Moorman, R. H.	1,538	Journal of Applied Psychology, 76(6), pp. 845-855, 1991
4	Equity, equality and need: What determines which value will be used as a basis of distributive justice?	Deutsch, M.	1,141	Journal of Social Issues, 31(3), pp. 137-149, 1975
5	Consequences of abusive supervision	Tepper, B.J.	1,095	Academy of Management Journal, 43(2), pp. 178-190, 2000
6	Antecedents and consequences of employee engagement	Saks, A. M.	1,086	Journal of Managerial Psychology, 21(7), pp. 600-619, 2006
7	Retaliation in the workplace: The roles of distributive, procedural and interactional justice	Skarlicki, D. P., Folger, R.	1,027	Journal of Applied Psychology, 82(3), pp. 434-443, 1997
8	The Role of Procedural Justice and Legitimacy in Shaping Public Support for Policing	Sunshine, J., Tyler, T. R.	929	Law and Society Review, 37(3), pp. 513-548+512, 2003
9	An integrative framework for explaining reactions to decisions: Interactive effects of outcomes and procedures	Brockner, J., Wiesenfeld, B.M.	761	Psychological Bulletin, 120(2), pp. 189-208, 1996
10	Affective commitment to the organization: The contribution of perceived organizational support	Rhoades, L., Eisenberger, R., Armeli, S.	741	Journal of Applied Psychology, 86(5), pp. 825-836, 2001

a *Rank, By most citations*.

b *Till 2018*.

Moreover, it is interesting to note that the 5,650 papers have total citations of 153,547. Among these, only 316 articles have more than 100+ citations, whereas 14% of the articles from 1941 to 2018 have never been cited. In addition, the citation range of the Table 3 articles is from 741 to 1,945. We disagree with Johnson's (2009) statement that older publications have more citations. In our analysis of Table 3, four articles belonged to twenty-first century.

### Leading Countries, Top Institutions, and International Collaboration

**Figure 5:**
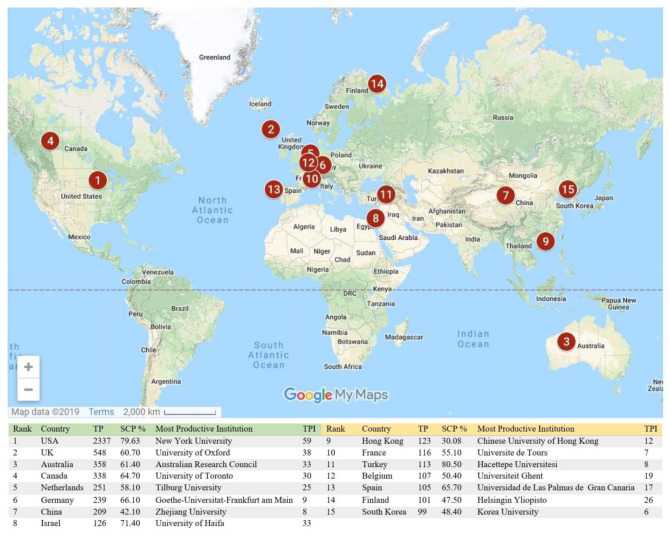
The top 15 countries and academic instituons on organizational justice research publications. TP, Total publications; SCP, Single-country publications; TPI, Total publications of institutions.

Figure 5 shows the top 15 countries contributing to the field of organizational justice. From 1941 to 2018, about 40% of the publications were contributed by the USA. Since the idea of organizational justice was first investigated by the American researchers in 1941, they have led the field. These 15 countries have produced 54.5% of the total publications on organizational justice research as single country publications. Nevertheless, from a total of 99 countries, 51 countries have fewer than 10 articles. One reason may be that most of these 15 top research countries belong to the advanced academic world, where the research culture is already developed. If we analyse these data by continent and regions wise, seven countries are from Europe, five are from Asia, two are from America and one is from Oceania region. No country is from Africa. Asia comprises 60% of the world population (World Population Review, 2019) and China is the most populated country in Asia and the world; the country secured seventh place on the list. Also, Africa is the second-highest continent, comprising 17% of the world's population, but no country from Africa was amongst the top 15. Yet Oceania region comprises just 0.55% of the world's population, but Australia was in third place.

Moreover, Figure 5 also highlights the contributions by country and the research affiliation with authors of other countries. Although the USA has the highest number of publications, but Turkey has more single country publications. In the same vein, the UK, Australia, Canada, Germany, and Spain have more than 60% of research projects with intra-country collaborations. In addition, Hong Kong, being a small territory has about 70% of its research projects with other country researchers. Similarly, China, Finland, and South Korea have more than 50% of its publications with researchers from other countries.

Furthermore, among the top institutions contributing the most to the field, 14 are universities and one is the research institute (Figure 5). This analysis shows that most of the research output is produced by university faculty members, research associates and students. Finally, among these 14 academic institutions, six are listed in the top 100 best universities based on the 2020 QS world university ranking (QS, 2019) as shown in Table 3: the University of Oxford (ranked 4th), the University of Toronto (ranked 29th), New York University (ranked 39th), the Chinese University of Hong Kong (ranked 46th), Zhejiang University (ranked 54th), and Korea University (ranked 83rd). The results demonstrate that organizational justice has received attention from the researchers at the top universities in the world. This analysis also shows that the top universities continue to lead in organizational justice research (Abramo et al., 2016). Among these, the University of Oxford, New York University and the Chinese University of Hong Kong are also among the sponsoring agencies of organizational justice research projects. Moreover, it also shows the scope of organizational justice research across the globe.

**Figure 6:**
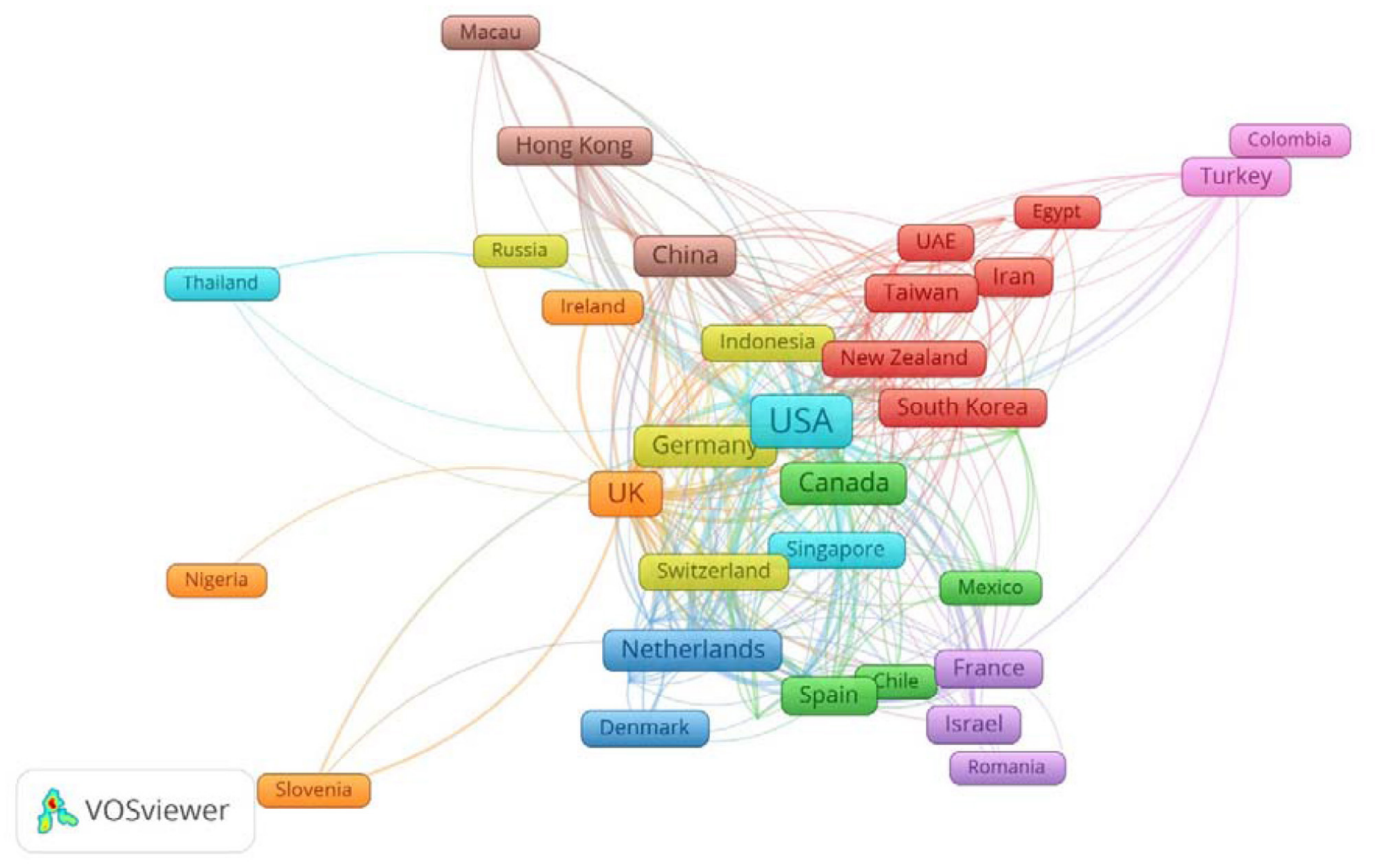
A snapshot of bibliometric map created based on co-afflifation of countries with network mode. The following URL can be utilized to open Figure 6 in VOSviewer: http://bit.ly/2oaN5S0.

The country network information demonstrates how authors of one country network with authors from other countries (Figure 6). The VOSviewer has allocated colors to countries on the basis of how much they network. Moreover, the closer the two countries are to each other, the stronger their relationship, as shown in Figure 6. In addition, the stronger the link between the two countries is, the thicker the line. If we interpret the data by continent, Asia (23 countries) is in first place, followed by Europe (20 countries), America (seven countries), Africa (four countries), and Oceania region (two countries). However, if we examine the number of countries in the continent and region and their appearance, Asia again leads with 52% of the countries, followed by Europe (42.55%), North America (20%), and Oceania region (14.28%). Not surprisingly, Africa has the highest number of countries among all the continents, but its total appearance is just 7%. Now and then, it is beneficial to have research ventures with different countries and particularly with developed countries. For example, authors in Hong Kong have network relationships with 17 countries (e.g., its link strength with USA = 33; with UK = 13; with Australia = 7), which is profoundly helpful. Hong Kong is now among the top 10 countries for research on organizational justice. Among the 55 countries mapped in VOSviewer, USA and the UK have highest number of co-authorship relationships with different countries, 47 each. However, the total number of co-authorship articles for the USA is 605; for the UK, it is 283. The citation number for these USA articles is 91,613; for the UK, it is 14,065. One reason of this large gap is the total number of publications (see Figure 5). It is also pertinent to mention here that Turkey has only 14 links with other countries authors and co-authorship of 30 articles because single country publications in Turkey account for about 80% of the total.

The high number of citations also shows that researchers acknowledge the work of USA authors. In addition, developing partnerships with researchers from other countries helps to widen research networks, share knowledge, develop relationships, and increase the profile of the researchers as well as their institution and country. This analysis is valuable for the researchers developing partnerships with researchers for research opportunities with different countries. Furthermore, as per Thelwall and Sud (2016) stated that more authors in an article increase citations of the articles. These results also reveal that new countries are publishing research articles in collaboration with other countries; this will pave the way to understand the cultural patterns of these countries in that particular research area.

### Leading Authors

The 10 most productive authors in organizational justice research are listed in Table 4. The total number of authors of these 5,650 articles is 13,430 whereas five articles authors undefined; the number of authors range from one author per article to 19. We set the threshold of five in the VOSviewer when we uploaded the data, which revealed 159 authors who had five or more articles. Furthermore, no author from this table is among the top six cited research articles on the Scopus database (see Table 3). Among the top 15 cited authors in the database for organizational justice research, only three authors are from Tyler, Cropanzano, and Folger. However, they also have co-authors in their articles. We also analyzed the data on the h-index and citations, since Egghe (2012) noticed that more than 50% articles published from 2007 to 2011 in the *Journal of Informetrics* were based on citations and/or the h-index.

**Table 4:** Top 10 highly published authors.

**Rank**	**Author**	**SAID^**a**^**	**YFP^**b**^**	**YRP^**c**^**	**TP^**d**^**	**h-index**	**TGC^**e**^**	**Affiliation**	**Country**
1	Tyler, Tom R.	55913409300	1977	2018	58	35	6,706	Yale University	USA
2	Elovainio, M.	7003614400	1995	2017	51	23	2,055	National Institute for Health and Welfare	Finland
3	Kivimaki, Mika	7004391239	1992	2017	39	23	2,234	University College London	England
4	Vahtera, Jussi	7003922524	1994	2017	32	20	1,832	Turun Yliopisto	Finland
5	Murphy, Kristina	7402861587	2003	2018	31	18	1,066	Griffith University	Australia
6	Cremer, D. De	7006810104	1998	2017	28	16	899	University of Cambridge	England
7	Cropanzano, R. S.	6603791691	1987	2017	24	16	3,018	The University of Colorado at Boulder	USA
8	Leung, Kwok	56664618400	1982	2016	23	14	1,076	Chinese University of Hong Kong	Hong Kong
9	Folger, Robert G.	7003324152	1974	2016	21	17	3,017	University of Central Florida	USA
10	Lind, E. A.	15752579100	1974	2014	21	16	1,978	Duke University	USA

a *SAID, Scopus Author ID*.

b *YFP, Year of first publication*.

c *YRP, Year of recent publication*.

d *TP, Total publications*.

e *TGC, Total global citations*.

Hirsch stated that an h-index over 40 indicates as outstanding researcher (Quoted by Ball, 2005) thus, no author has an h-index over 40 (see Table 4). However, these authors' number of publications, citations and h-index is quite high, but we collected data only for organizational justice publications. Similarly, Cropanzano and Folger are low in terms of total publications, but their citations are quite high as compared to other authors. Moreover, these top 10 authors published 5.8% of the total publications on organizational justice research. Furthermore, these top 10 highly productive authors are affiliated with five countries; the USA leads with four out of the 10 most productive authors.

We also compared top journals (Table 2), highly cited articles (Table 3), and most productive authors (Table 4) with each other. The comparison of these tables revealed the information that highly productive authors have highly cited articles in the top journals. Our outcomes reveal that highly published authors Folger and Tyler also have articles in Table 3. In addition, both have co-authors in their articles also. Furthermore, the top 10 authors also have publications with other authors. In any case, this does not question the top 10 highly productive author's number of publications in most productive journals or high citation score. For instance, Tyler has article with a citation score of 927; however, that journal is not in the top 10 of exceptionally productive journals, as referenced in Table 2. Moreover, these top 10 scholars also have publications in the journals listed in Table 2. Our findings show, as in Table 4, that Folger and Cropanzano have articles, ranked 5 and 7 (Table 2), respectively, and these are highly cited articles of these journals.

As indicated by Md Khudzari et al. (2018), author names in the research articles have no appropriate succession, and the last position is typically connected with senior author. We concur with the first statement while not with the second (see Tyler and De Cremer, 2005; De Cremer and Tyler, 2007). Yet, the appearance of authors name in the journals is the mutual understanding of the authors contributed in an article.

### Analysis of Organizational Justice and Its Dimensions

**Figure 7:**
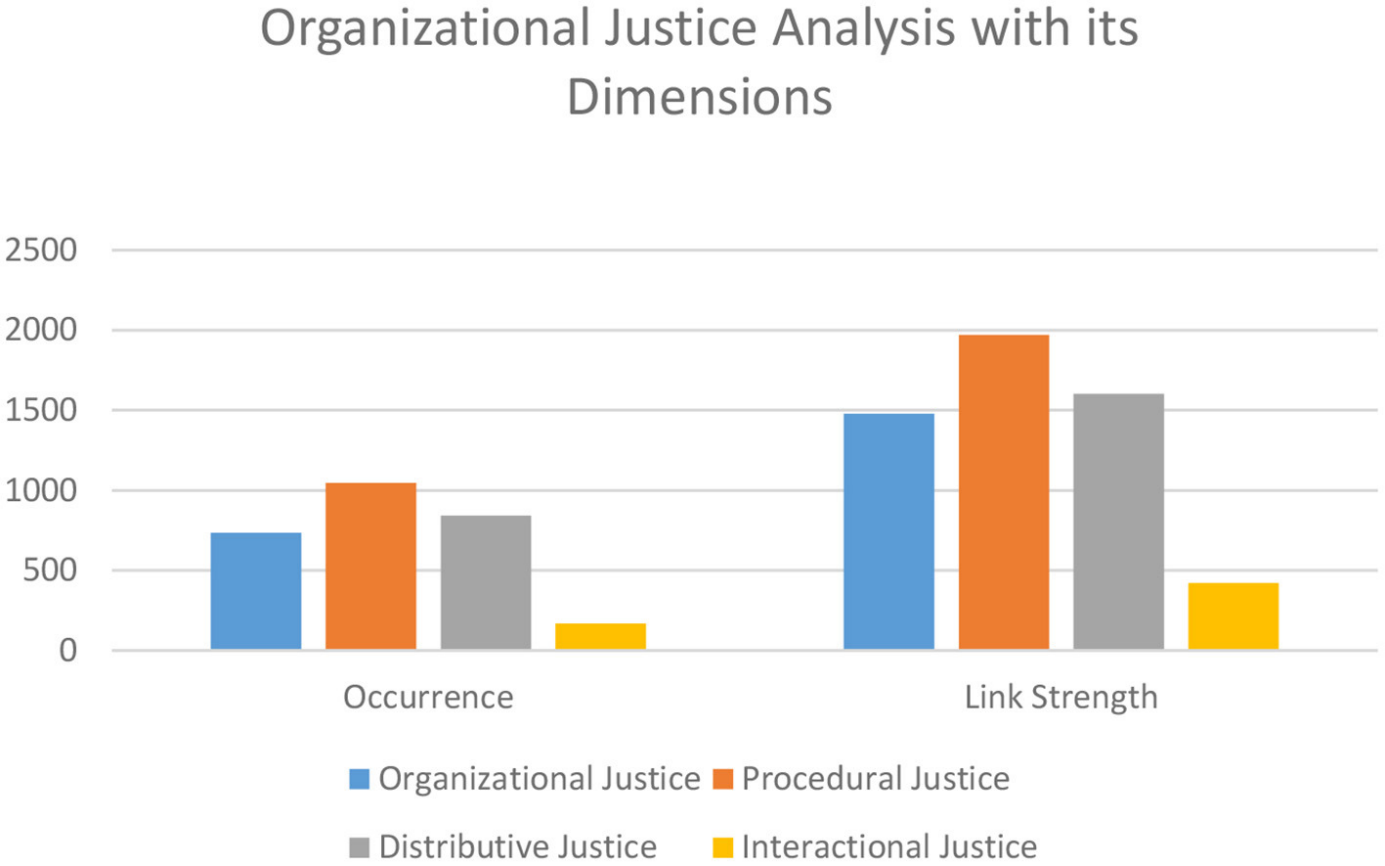
Organizational justice and its dimensions analysis with link strength.

The results show that the term “organizational justice;” has *n* = 736 and 1,477 total link strength to other keywords (Figure 7). “Procedural justice” is the most frequently encountered keyword with *n* = 1,047, and 1,969 total link strength to other keywords followed by “distributive justice” with *n* = 842 and 1,602 total link strength to other keywords. The data also show a strong link between procedural justice and distributive justice, with *n* = 222. Moreover, the third dimension of justice, i.e., interactional justice, occurs less frequently. The total occurrences of interactional justice were 168, with 421 link strength to other keywords, which shows that interactional justice along with its two sub-dimensions—interpersonal justice (*n* = 52, total links: 135) and informational justice (*n* = 36, total links: 124)—occur infrequent in the organizational justice realm. The results also endorse the findings of the Rupp et al. (2017) and Pan et al. (2018) who highlight that organizational justice has two main dimensions: procedural justice and distributive justice. From 1941 to 1987, the dimension of distributive justice dominated. In 1982, Melton and Lind talked categorically about procedural justice. Now, among these two, the most dominating is procedural justice, which is supported by our data and also by the data of Zoghbi-manrique-de-lara and Ting-ding (2017). Because, fair procedures satisfy employees even when the distribution does not (Lind and Tyler, 1988). In addition, Cropanzano et al. (2016) stated that people give special attention to procedural justice. However, Raja et al. (2018) highlights that in developing countries, distributive justice is more significant than procedural justice. Furthermore, no other dimension of organizational justice was found in our search. Besides, “overall justice” has only *n* = 10, which verifies the previous researchers' arguments that there are limited studies on the concept of overall justice (Ambrose et al., 2015). It also provides food for thought for new research projects.

#### Analysis of Organizational Justice With Other Keywords

In terms of keywords analysis, 29% of the articles were without author keywords. Moreover, we defined five as the minimum number of keyword appearances when uploading the data in the VOSviewer (Van Eck and Waltman, 2019). Thus, the database identified 390 keywords after finding synonyms for the 8,496 keywords. These 390 keywords have 4,470 links with each other, whereas the total link strength reaches to 10,786. We performed further analysis to see the relationship of organizational justice and its dimensions with other organizational variables, such as organizational commitment, job satisfaction, organizational trust, organizational citizenship behavior, social exchange theory, and others (see Table 5). We also noticed while reviewing the articles that the words “justice” and “fairness” were used interchangeably (see also Rego et al., 2009; Colquitt et al., 2013; Hillebrandt and Barclay, 2013; Gyekye and Haybatollahi, 2015; Zoghbi-manrique-de-lara and Ting-ding, 2017; Wolfe et al., 2018) but we considered these as separate entities to see their strength (see Goldman and Cropanzano, 2015). Justice has *n* = 505, whereas fairness has *n* = 248; both of the terms are linked more with distributive justice. It is pertinent to mention here that justice and fairness are also linked with each other, with a link strength of 52.

**Table 5:** Top 10 most occurred keywords.

**Rank**	**Variable**	**Occurrence**	**Link**	**TLS^a^**	**Organizational justice/ dimensions**	**LS^**b**^**
1	Organizational Trust	192	121	486	Procedural Justice	71
2	Organizational Commitment	186	91	480	Organizational Justice	83
3	Job Satisfaction	183	104	457	Organizational Justice	87
4	Organizational Citizenship Behavior	167	96	422	Organizational Justice	80
5	Ethics	98	84	178	Distributive Justice	25
6	Turnover	78	64	217	Organizational Justice	33
7	Job Performance	68	64	166	Procedural Justice	17
8	Justice Climate	61	59	107	Procedural Justice	13
9	Social Exchange Theory	60	60	148	Organizational Justice	22
10	Police Legitimacy	59	30	113	Procedural Justice	52

a *TLS, Total link strength*.

b *LS, Link strength*.

Table 5 also shows the most occurrences of the various keywords' relationship with organizational justice in Scopus database. The term “organizational trust” has secured the top place, whereas organizational commitment, job satisfaction and organizational citizenship behavior have secured the second, third, and fourth place, respectively (Colquitt et al., 2013). One reason that organizational trust occurs highly is because organizational justice is considered as a source of organizational trust (Dirks and Ferrin, 2002). The results also reveal that organizational trust is highly linked (71 link strength) with procedural justice, as compared to other justice dimensions, which also supports the study of McFarlin and Sweeney (1992). Keywords such as “ethics” (*n* = 98), “turnover” (*n* = 78), “job performance” (*n* = 68), and “climate justice” (*n* = 61) are also among the most frequent ones used. We have also noticed that some of the critical concepts, such as motivation, do not have many occurrences, which highlight areas for future research projects.

**Figure 8:**
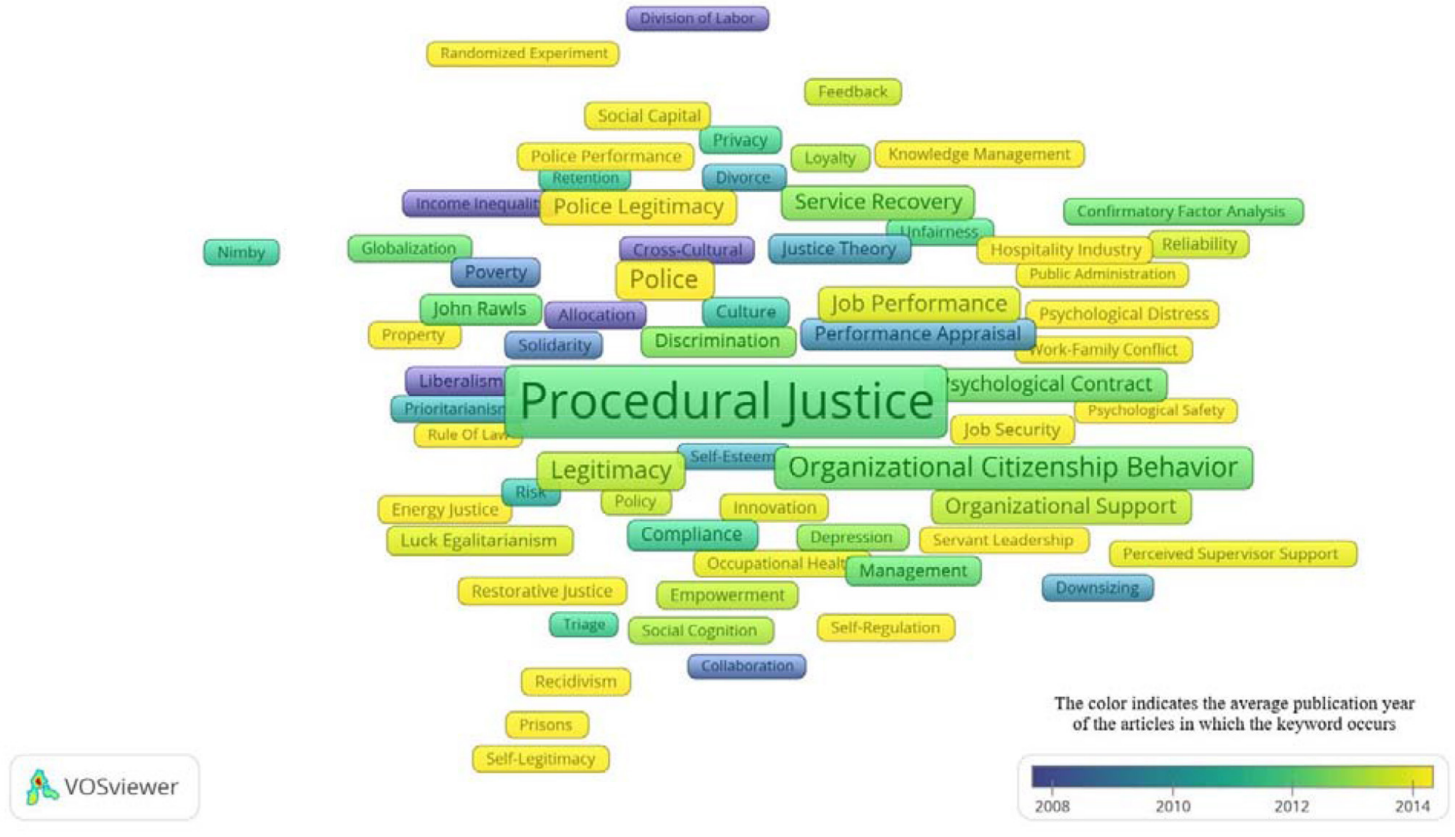
A snapshot of bibliometric map of authors keywords with overlay utilization mode. The following URL can be utilized to open Figure 8 in VOSviewer: http://bit.ly/2OiZ9eF.

Figure 8 shows the overlay visualization mode, which is represented with various colors. The overlay visualization mode shows the average publication year of the keywords. For example, yellow color represents the variables with average publication year 2014 whereas purple color represents the variables average publication year 2008. Moreover, this color scheme also shows the variables with infrequent occurrences. For instance, authentic leadership (*n* = 5), knowledge management (*n* = 7), psychological safety (*n* = 5), workplace bullying (*n* = 7), social media (*n* = 8), psychological distress (*n* = 12), corporate social responsibility (*n* = 21), job burnout (*n* = 38), organizational identification (*n* = 28), ethical leadership (*n* = 12), counterproductive work behavior (*n* = 15), work-family conflict (*n* = 7), job stress (*n* = 19), and others all have been displayed with yellow color. The overlay visualization mode also identifies variables by their publication years which have fewer occurrences with organizational justice studies. However, more research is needed to find out the influence of organizational justice on such variables as service fairness (*n* = 5), consumer behavior (*n* = 9), social psychology (*n* = 12), and group value model (*n* = 11).

#### Topics of Interest

Organizational justice is considered one of the significant variable in the realm of the organizations. It has been studied in every position in various research frameworks such as predictor variable, mediator, moderator, and criterion variable. Moreover, it has also been studied as uni-dimensional, multi-dimensional and overall justice concept which shows the versatility of the variable.

If some of the fundamental concepts of organizational behavior are grouped, it provides functional analysis relevant to organizational justice, which also establishes the theme of organizational justice research. These fundamental concepts are both the antecedents and consequences of organizational justice. We identified four significant keywords groups: leadership-related, job-related, organization-related, and social-related.

**Figure 9:**
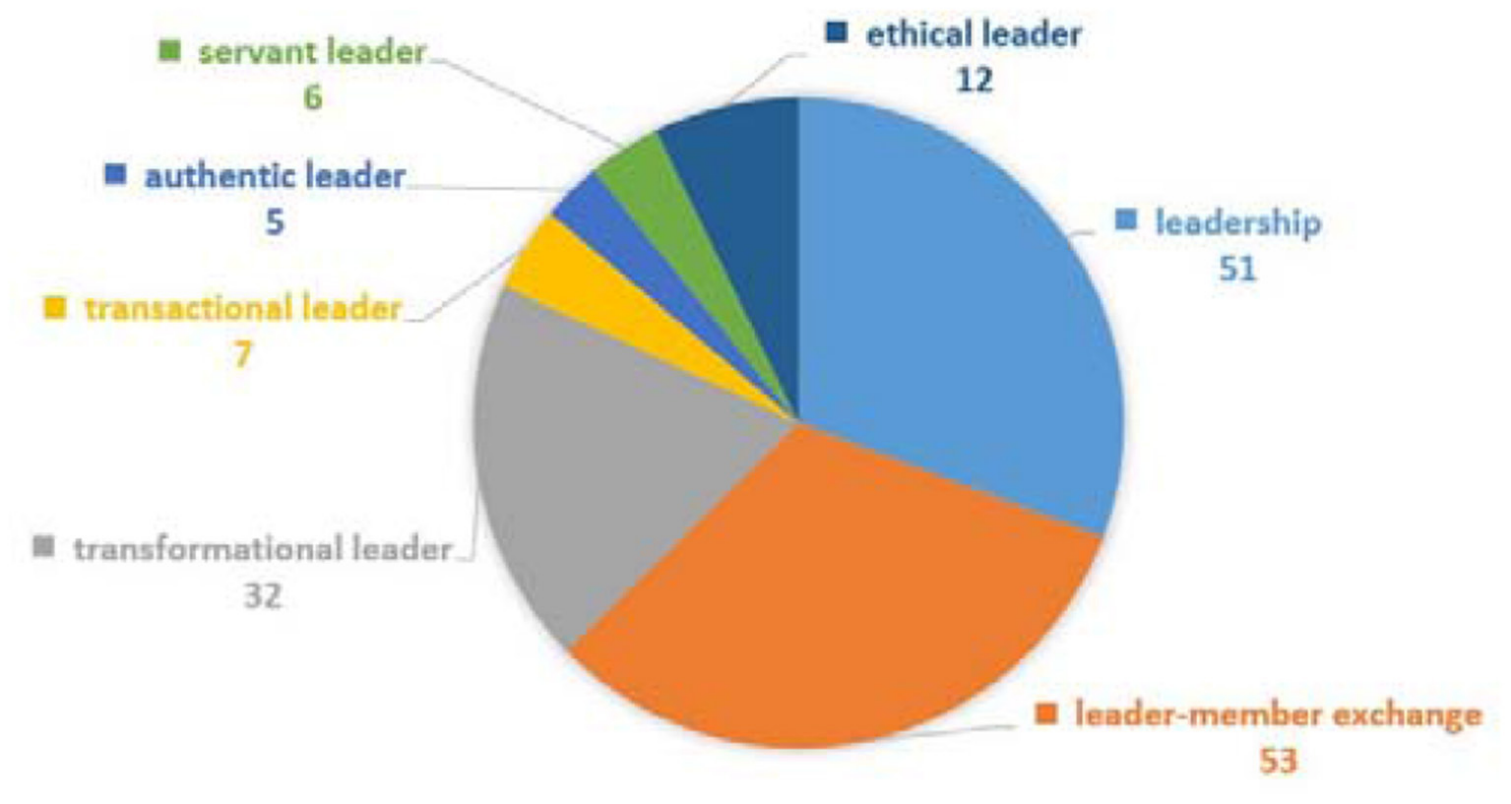
Leadership-related keywords analysis.

**Figure 10:**
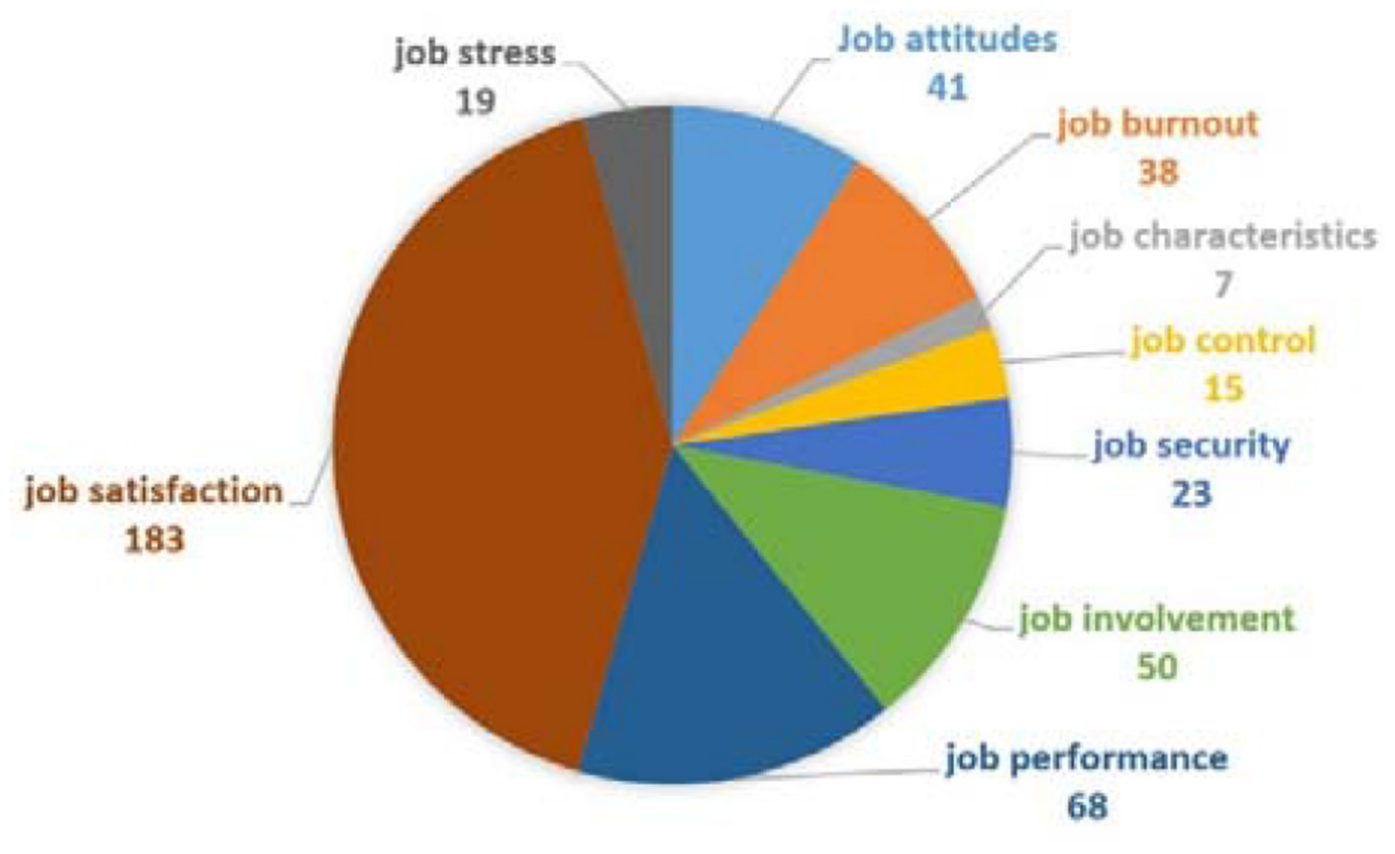
Job-related keywords analysis.

**Figure 11:**
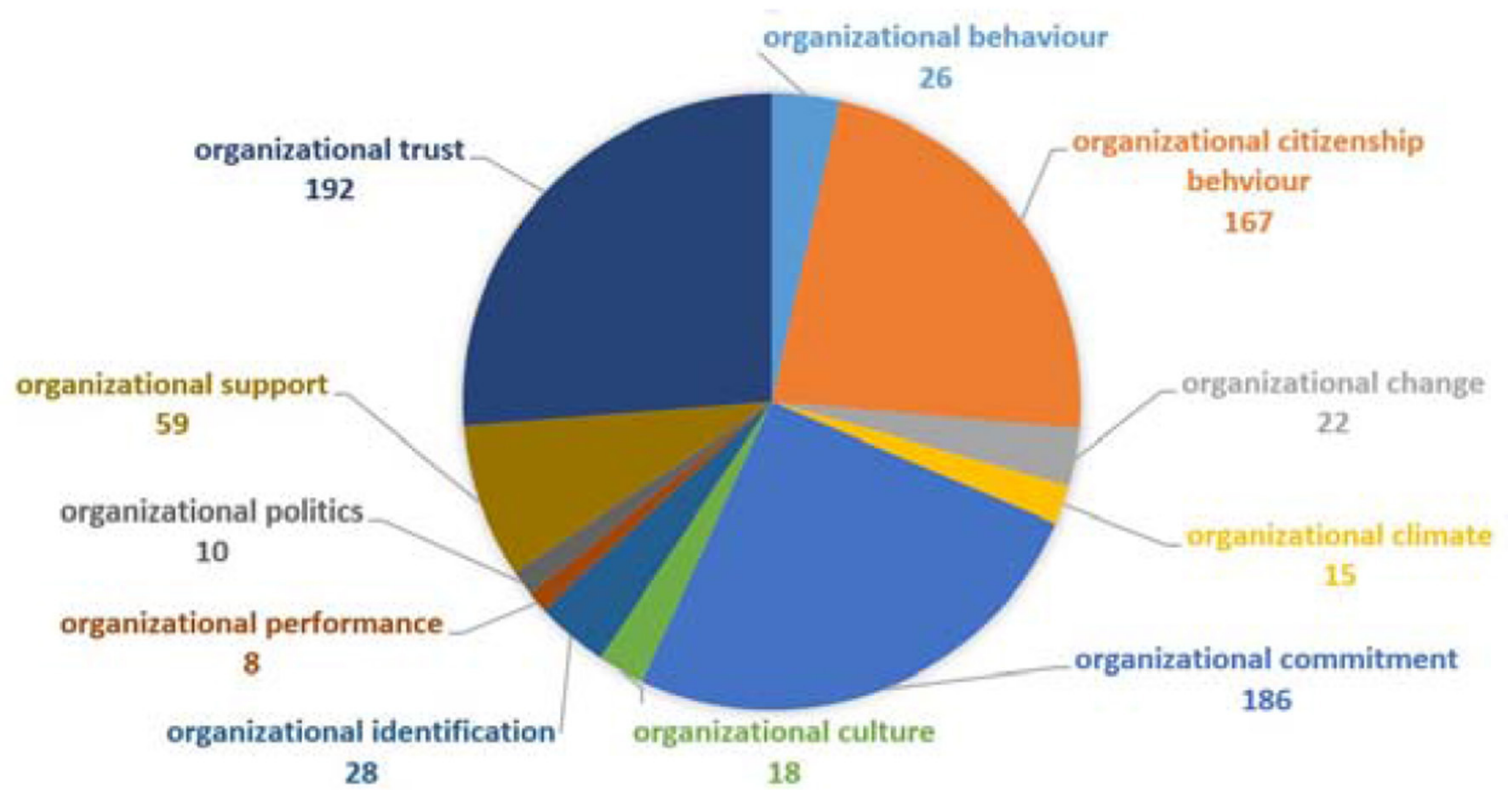
Organization-related keywords analysis.

The first group is leadership-related keywords analysis along with its types. Tyler and Caine (1981) first presented the role of “formal leader” in the field of organizational justice. Afterwards, various studies were conducted with different forms of leadership. The total occurrence of this group is 166. Except for LMX (*n* = 53) and transformational leadership (*n* = 32), all of the other types of leadership have fewer occurrences, as shown in Figure 9—for example, authentic leader (*n* = 5), servant leader (*n* = 6), and transactional leader (*n* = 7). We noted that both transformational and transactional leadership styles used to study with organizational justice in 1999 (Bass and Steidlmeier, 1999; Pillai et al., 1999), but gradually number of studies increased with transformational leadership styles whereas very rare with transactional leadership. On the basis of infrequent occurrences of transactional leadership in the past decade, we predict that this style of leadership has fewer occurrences in coming years also, whereas, more occurrences of the rest of leadership styles can be expected in near future. Although leadership is a significant variable in the field of organizational justice because leaders are at the justice giving end (Cunningham and Cordeiro, 2003), the occurrences of leadership styles are infrequent with organizational justice field. Moreover, the occurrences of leadership in organizational justice research are also lacking compared to other variables, such as job satisfaction and organizational trust (see Figures 10, 11, respectively).

The second group is job-related keywords analysis related to articles published on organizational justice (Figure 10). This group includes many key concepts of organizational behavior, such as job satisfaction (*n* = 183), job performance (*n* = 68), and job involvement (*n* = 50). Among these, the job satisfaction and job performance gained much recognition (Ambrose and Schminke, 2009; Kozlowski, 2017) and both these concepts emerged noticeably in 1987. Kanfer (1987) studied the justice effects on work performance whereas Greenberg (1987) discussed the role of justice in performance appraisal. On the other hand, Blegen and Mueller (1987) presented a longitudinal study on nurses' job satisfaction. We noted that organizational justice effects job performance but number of occurrences of job performance are less than the other variables such as citizenship behavior, job satisfaction, organizational trust, and organizational commitment. One reason may be that these variables indirectly contribute in the job performance. Moreover, surprisingly, there are fewer occurrences of job stress (*n* = 19), job security (*n* = 23), and job control (*n* = 15). However, the total occurrences of this group are 444.

Organizational related keywords are also the leading keywords group with total keywords occurrence (*n* = 731) and holds a significant importance in the studies related to organizational performance. These keywords have organization-related keywords analysis, with total occurrences (*n* = 731). However, this group consists of very functional concepts (see Figure 11). This group can be classified further into three categories: high occurrences, normal and few occurrences and organizational trust (*n* = 192), citizenship behavior (*n* = 167), and commitment (*n* = 186) are the highly occurred keywords. Most important, these three keywords are the important outcomes of an organization performance which are affected by organizational justice or its dimensions (Colquitt et al., 2001). Organizational support keyword occurs normally (*n* = 59). According to Cropanzano et al. (2016) these variables act reciprocally with organizational justice. If organizational justice is high, these variables would also be high and vice versa.

In addition, organizational performance (*n* = 8), organizational culture (*n* = 18), organizational climate (*n* = 15), organizational identification (*n* = 28), organizational change (*n* = 22), and organizational politics (*n* = 10) are also important keywords but have low occurrence in this study. Among all these, the occurrence of organizational performance is quite surprising and unexpected. As Colquitt et al. (2001) stated that organizational performance is an important outcome of organizational justice. Two plausible reasons can be argued for this low occurrence. First, other variables such as citizenship behavior, commitment, satisfaction, support and trust also contribute toward performance and researchers are studying these variables separately. Second, we separated the job performance with organizational performance.

**Figure 12:**
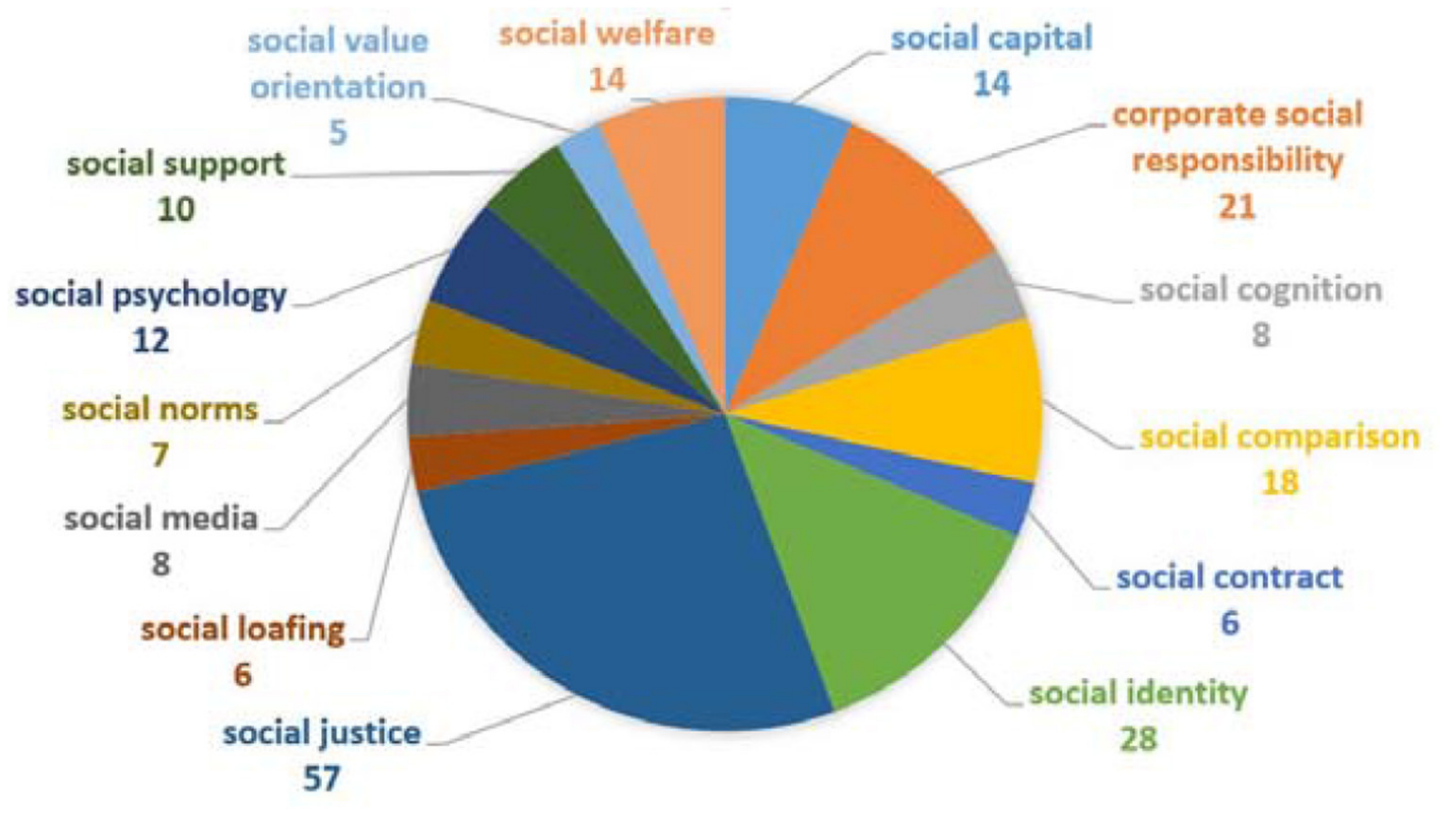
Social-related keywords analysis.

The fourth and largest keyword group is social-related keywords analysis (see Figure 12). This group has a total of 14 keywords with a total occurrence 214. However, instead of social justice (*n* = 58), rest of other keywords have <30 occurrences. One reason for low occurrences of these keywords may be caused by lesser importance or insignificant influence on organizational performance. But this group has some significant variables, which have started to receive attention from researchers, such as corporate social responsibility research with organizational justice (see Sarfraz et al., 2018; Farid et al., 2019).

Among all the theories, Blau's (1964) social exchange theory (SET) occurs the most. Bagarozzi and Wodarski (1977) are among the early authors who used SET with distributive justice. Colquitt et al. (2013) refer to the study of Organ and Konovsky (1989), which first used SET to understand the relationship between organizational justice and organizational citizenship behavior. The total occurrences of SET were 60, with a link strength of 148. Moreover, SET has a strong link with organizational justice (*n* = 22) as compared to procedural justice (*n* = 11), distributive justice (*n* = 4), and interpersonal justice (*n* = 3). This analysis also reveals that SET occurs with all the dimensions of organizational justice. Rawls' (1971) theory of justice is the second highest (*n* = 18) whereas Deci and Ryan's (1985) self-determination theory is in third place (*n* = 15) and Adams's (1965) equity theory is in fourth place (*n* = 12).

Furthermore, uncertainty management theory (*n* = 7) and affective events theory (Weiss and Cropanzano, 1996) (*n* = 5) are in fifth and sixth place, respectively. Thus, SET has secured a firm footing within the field of organizational justice as compared to other theories. Moreover, among various models to study organizational justice, the group value model (Lind and Tyler, 1988) has 11 occurrences; among these, eight occurrences are for procedural justice, whereas only three occurrences are for distributive justice.

## Implications

The current bibliometric study will make a major contribution to research on organizational justice by demonstrating a wide range of statistical analyses and critically examining the trends and scope of the field of organizational justice since 1941, the year of the first article on organizational justice indexed in the Scopus database. More precisely, following are the major implications for the researchers:

a. Results reveal that researchers have shown more interest in procedural justice as compared to other dimensions of organizational justice.
b. Results also provide food for thought to conduct more studies to understand the phenomena better.
c. Organizational justice research is moving more toward organizational trust, citizenship behavior, organizational commitment, and job satisfaction.
d. More research is needed to find out the influence of organizational justice on variables such as service fairness, consumer behavior, social psychology, and group value model.
e. Researchers should develop the research linkages with most productive authors and advanced countries so that their portfolio may also increase.
f. Researchers may use various leadership styles to study with organizational justice.

## Limitation and Future Recommendations

Despite all the efforts and time poured in this research, it does have some limitations. First, only organizational justice and its dimensions were used to explore the database; thus, articles using terms such as “fair” or “fairness” were not included, even though they may refer to organizational justice. The present articles intentionally did not include these terms, as the results were beyond the scope of the present analysis. Future researchers may want to include these terms for a more comprehensive analysis of the variable, but this may limit the research to a particular subject area. As this study was an attempt to include all the variables among the subject areas listed in the Scopus database, it did not constrain itself to any particular field. Moreover, it is hard to apply the findings to any particular field; instead, the researchers of every field can take the initial ideas for future endeavors.

Second, the co-occurrence analysis of author's keywords covered only 70.97% of the articles due to missing author keyword information from certain journals. Therefore, our analysis is limited to these author's keywords. Moreover, the journals without author's keywords are too many in number, and they cannot be included here. Third, the Scopus database does not provide any systematic search string, and the we did not try to explore whether organizational justice has been explored mostly as an independent variable or as mediator/moderator one. Although mediator and moderator keywords appeared in the database, we are firmly convinced that these occurrences do not cover the full range of the 5,650 articles. The main reason may be that studies hardly use the words of moderator or mediator in their title, abstract and keywords. The best solution for this problem is to read every article one by one. Thus, we recommend for future researchers to cover this aspect as well. Fourth, another aspect which is not covered in this study is the research design: qualitative, quantitative, or mix method. The results of the database show limited results for quantitative and qualitative studies, but again this is not verified and may be addressed by future researchers. Lastly, the bibliometric analysis on organizational justice was till 2018. The future researchers may extend this study results till 2020 and from other databases such as Journal Citation Report.

## Conclusion

We have analyzed the organizational justice research indexed in the Scopus database through mapping with authors, keywords and countries. In turn, this analysis reveals that organizational justice matters for organizations all over the world. Moreover, researchers' interest in the topic has increased in recent years. In addition, social science scholars, as well as managers and psychologists, publish most of the research in the field. Although developed countries dominate the field in both number of publications and productive authors, developing countries have begun to contribute as well. Furthermore, researchers have shown more interest in procedural justice than distributive and international justice. We have also presented the trend of the publications with well-known keywords as well as the overlooked ones. We attempted to explain the past research trends and major practicing keywords but found no clear theme except that organizational justice research is moving more toward organizational trust, citizenship behavior, organizational commitment, and job satisfaction. It is likely that since the knowledge world is growing, some key concepts currently dominating the field now may be replaced with more influential keywords in the future.

## Data Availability Statement

The raw data supporting the conclusions of this article will be made available by the authors, without undue reservation.

## Author Contributions

All authors listed have made a substantial, direct and intellectual contribution to the work, and approved it for publication.

## Conflict of Interest

The authors declare that the research was conducted in the absence of any commercial or financial relationships that could be construed as a potential conflict of interest.
