# Supplementary material for: The Research on Organizational Justice in Scopus Indexed Journals: A Bibliometric Analysis of Seven Decades
Source: Front Psychol. 2021 Jun 10;12:647845. doi: 10.3389/fpsyg.2021.647845 (PMC8222511; doi:10.3389/fpsyg.2021.647845)
Supplement: Supplementary file 1 [file Data_Sheet_1.docx]

Appendix A

| Sr | Search Purpose | Search String |
| --- | --- | --- |
| 1. | Articles (main search) | TITLE-ABS-KEY ( "Organizational Justice" )  OR  TITLE-ABS-KEY ( "Procedural Justice" )  OR  TITLE-ABS-KEY ( "Distributive Justice" )  OR  TITLE-ABS-KEY ( "Interactional Justice" )  OR  TITLE-ABS-KEY ( "Interpersonal Justice" )  OR  TITLE-ABS-KEY ( "Informational Justice" ) |
| 2. | Excluded 2019-2020 articles from main search | TITLE-ABS-KEY ( "Organizational Justice" )  OR  TITLE-ABS-KEY ( "Procedural Justice" )  OR  TITLE-ABS-KEY ( "Distributive Justice" )  OR  TITLE-ABS-KEY ( "Interactional Justice" )  OR  TITLE-ABS-KEY ( "Interpersonal Justice" )  OR  TITLE-ABS-KEY ( "Informational Justice" )  AND  ( EXCLUDE ( PUBYEAR ,  2020 )  OR  EXCLUDE ( PUBYEAR ,  2019 ) ) |
| 3. | Limited to research articles published in Journals only (from main search) | TITLE-ABS-KEY ( "Organizational Justice" )  OR  TITLE-ABS-KEY ( "Procedural Justice" )  OR  TITLE-ABS-KEY ( "Distributive Justice" )  OR  TITLE-ABS-KEY ( "Interactional Justice" )  OR  TITLE-ABS-KEY ( "Interpersonal Justice" )  OR  TITLE-ABS-KEY ( "Informational Justice" )  AND  ( LIMIT-TO ( SRCTYPE ,  "j" ) )  AND  ( LIMIT-TO ( DOCTYPE ,  "ar" ) )  AND  ( EXCLUDE ( PUBYEAR ,  2020 )  OR  EXCLUDE ( PUBYEAR ,  2019 ) ) |
| 4. | Identify review articles | TITLE-ABS-KEY ( "Organizational Justice" )  OR  TITLE-ABS-KEY ( "Procedural Justice" )  OR  TITLE-ABS-KEY ( "Distributive Justice" )  OR  TITLE-ABS-KEY ( "Interactional Justice" )  OR  TITLE-ABS-KEY ( "Interpersonal Justice" )  OR  TITLE-ABS-KEY ( "Informational Justice" )  AND  ( TITLE ( "recent"  OR  progress  OR  meta-analysis  OR  meta-analytic  OR  review  OR  critical  OR  revisit  OR  advance  OR  development  OR  highlight  OR  perspective  OR  prospect  OR  trends  OR  bibliometric  OR  scientometric )  OR  ( ABS ( progress  OR  review  OR  bibliometric  OR  scientometric ) ) )  AND  ( LIMIT-TO ( SRCTYPE ,  "j" ) )  AND  ( LIMIT-TO ( DOCTYPE ,  "ar" ) )  AND  ( EXCLUDE ( PUBYEAR ,  2020 )  OR  EXCLUDE ( PUBYEAR ,  2019 ) ) |
| 5. | Removed all review articles from main search | TITLE-ABS-KEY ( "Organizational Justice" )  OR  TITLE-ABS-KEY ( "Procedural Justice" )  OR  TITLE-ABS-KEY ( "Distributive Justice" )  OR  TITLE-ABS-KEY ( "Interactional Justice" )  OR  TITLE-ABS-KEY ( "Interpersonal Justice" )  OR  TITLE-ABS-KEY ( "Informational Justice" )  AND NOT EID (List of Review Articles* )  AND  ( LIMIT-TO ( SRCTYPE ,  "j" ) )  AND  ( LIMIT-TO ( DOCTYPE ,  "ar" ) )  AND  ( EXCLUDE ( PUBYEAR ,  2020 )  OR  EXCLUDE ( PUBYEAR ,  2019 ) ) |

*List of 361 EID is available with the authors and can be provided on request.

Appendix B

Top Journals with highest CiteScore more than 20 articles on Organizational Justice

| Rank | Journal | CiteScore | Journals Homepage | Publisher | TP* |
| --- | --- | --- | --- | --- | --- |
| 1 | Journal of Management | 10.96 | <https://journals.sagepub.com/home/jom> | SAGE | 35 |
| 2 | Academy of Management Journal | 10.36 | <http://aom.org/amj/> | Academy of Management | 25 |
| 3 | Journal of Personality and Social Psychology | 7.41 | <https://www.apa.org/pubs/journals/psp/> | APA | 34 |
| 4 | Personnel Psychology | 7.1 | <https://onlinelibrary.wiley.com/journal/17446570> | Wiley-Blackwell | 21 |
| 5 | Journal of Applied Psychology | 6.86 | <https://www.apa.org/pubs/journals/apl/> | APA | 106 |
| 6 | Journal of Organizational Behavior | 6.59 | <https://onlinelibrary.wiley.com/journal/10991379> | Wiley-Blackwell | 63 |
| 7 | Journal of Business Research | 5.32 | <https://www.sciencedirect.com/journal/journal-of-business-research> | Elsevier | 28 |
| 8 | Journal of Criminal Justice | 4.8 | <https://www.sciencedirect.com/journal/journal-of-criminal-justice> | Elsevier | 22 |
| 9 | Journal of Business Ethics | 4.46 | <https://www.springer.com/philosophy/ethics+and+moral+philosophy/journal/10551?cm_mmc=sgw-_-ps-_-journal-_-10551> | Springer Nature | 91 |
| 10 | Human Relations | 4.24 | <https://journals.sagepub.com/home/hum> | SAGE | 24 |
| 11 | Organizational Behaviour and Human Decision Processes | 3.82 | <https://www.sciencedirect.com/journal/organizational-behavior-and-human-decision-processes> | Elsevier | 52 |
| 12 | European Journal of Work and Organizational Psychology | 3.61 | <https://www.tandfonline.com/toc/pewo20/current> | Taylor & Francis | 24 |
| 13 | Journal of Experimental Social Psychology | 3.5 | <https://www.sciencedirect.com/journal/journal-of-experimental-social-psychology> | Elsevier | 27 |
| 14 | Law and Human Behavior | 3.32 | <https://www.apa.org/pubs/journals/lhb/> | APA | 22 |
| 15 | Group and Organization Management | 3.31 | <https://journals.sagepub.com/home/gom> | SAGE | 26 |
| 16 | Journal of Business and Psychology | 3.17 | <https://www.springer.com/psychology/journal/10869?cm_mmc=sgw-_-ps-_-journal-_-10869> | Springer Nature | 37 |
| 17 | Criminal Justice and Behavior | 2.76 | <https://journals.sagepub.com/home/cjb> | SAGE | 31 |
| 18 | International Journal of Human Resource Management | 2.71 | <https://www.tandfonline.com/toc/rijh20/current> | Taylor & Francis | 54 |
| 19 | Journal of Managerial Psychology | 2.05 | <http://www.emeraldgrouppublishing.com/products/journals/journals.htm?id=JMP> | Emerald | 43 |
| 20 | Journal of Applied Social Psychology | 1.99 | <https://onlinelibrary.wiley.com/journal/15591816> | Wiley-Blackwell | 55 |
| 21 | Personnel Review | 1.95 | <http://www.emeraldgrouppublishing.com/products/journals/journals.htm?id=PR> | Emerald | 33 |
| 22 | Policing and Society | 1.88 | <https://www.tandfonline.com/loi/gpas20> | Taylor & Francis | 22 |
| 23 | Policing | 1.76 | <http://www.emeraldgrouppublishing.com/products/journals/journals.htm?id=pij> | Emerald | 31 |
| 24 | International Journal of Conflict Management | 1.58 | <http://www.emeraldgrouppublishing.com/products/journals/journals.htm?id=IJCMA> | Emerald | 30 |
| 25 | Social Justice Research | 1.57 | <https://www.springer.com/psychology/personality+%26+social+psychology/journal/11211?cm_mmc=sgw-_-ps-_-journal-_-11211> | Springer Nature | 155 |
| 26 | Journal of Medical Ethics | 1.44 | <https://jme.bmj.com/> | BMJ Publishing Group | 24 |
| 27 | Journal of Applied Philosophy | 1.23 | <https://onlinelibrary.wiley.com/journal/14685930> | Wiley-Blackwell | 21 |
| 28 | Politics, Philosophy and Economics | 1.13 | <https://journals.sagepub.com/home/ppe> | SAGE | 22 |
| 29 | International Journal of Selection and Assessment | 1.06 | <https://onlinelibrary.wiley.com/journal/14682389> | Wiley-Blackwell | 27 |
| 30 | Employee Responsibilities and Rights Journal | 0.81 | <https://link.springer.com/journal/10672> | Springer Nature | 27 |
| 31 | Social Behavior and Personality | 0.8 | <https://www.sbp-journal.com/index.php/sbp> | Society for Personality Research | 25 |

*TP: Total Publications
